# Genetic association between the cyclin-dependent kinase inhibitor gene *p27/Kip1* polymorphism (rs34330) and cancer susceptibility: a meta-analysis

**DOI:** 10.1038/srep44871

**Published:** 2017-03-20

**Authors:** Xiao-Ke Cheng, Xue-Jun Wang, Xiao-Dong Li, Xue-Qun Ren

**Affiliations:** 1 Center for Evidence-Based Medicine, Huaihe Hospital of Henan University, Kaifeng, Henan, 475000, China; 2 Department of Emergency, Beijing Electric Power Hospital, Taipingxili Jia 1, Beijing, 100073, China; 3 Department of Urology, Huaihe Hospital of Henan University, Kaifeng, Henan, 475000, China; 4 Department of General Surgery, Huaihe Hospital of Henan University, Kaifeng, Henan, 475000, China

## Abstract

The *p27* rs34330 (-79C/T) polymorphism has been widely studied for human cancer susceptibility. The current findings, however, still remained controversial. Therefore, we performed the meta-analysis to provide a more accurate result. Eligible studies were identified from PubMed database up to June 2015. The association of *p27* rs34330 polymorphism and cancer susceptibility was estimated with odds ratios and corresponding 95% confidence intervals. The meta-analysis was performed with Stata 12. A total of ten studies with 11,214 cases and more than 8,776 controls were included in the meta-analysis (including breast, lung, thyroid, endometrial, and hepatocellular cancer). In pooled analysis, *p27* gene rs34330 polymorphism significantly increased the cancer susceptibility. Subgroup analysis indicated that the elevated risk was observed under all the genetic models for Asians and under three genetic models for Caucasians. Results of sensitivity analysis were similar to the overall results. The results suggested that the *p27* rs34330 polymorphism increased the cancer susceptibility, especially in Asians. Further well-designed and large sample size studies are warranted to verify the conclusion.

Cancer is a leading cause of death and a major public health problem in both economically developed and developing countries. The occurrence of cancer is elevating because of the growth and aging of global population and environmental factors, especially in less developed countries, in which approximately 82% of the world’s population resides. Based on GLBOCAN estimates, about 14.1 million new cancer cases and 8.2 million deaths occurred in 2012 worldwide^1^. Many risk factors, such as lifestyle behaviors^2^,^3^,^4^ and genetic factors^5^,^6^,^7^,^8^,^9^, have been identified. However, cancer prevention is still a challenging project. Therefore, it is urgent to identify other risk factors for preventing cancers.

The *p27/Kip1 (p27*) gene (also known as *CDKN1B*) is located on chromosome 12p13 and encodes cyclin-dependent kinase inhibitors (CDKIs) implicated in the negative regulation of the cell cycle^10^,^11^. Cell cycle arrest allows cells to repair DNA damage and replication errors. Therefore, the loss of cell cycle control may contribute to the development of malignancies^12^,^13^. Certain polymorphisms including rs2066827 (109T/G) and rs34330 (-79 C/T) of *p27* gene have been identified as associated cancer susceptibility. In 2012, a meta-analysis had been performed to estimate the association between *p27* gene rs2066827 polymorphism and cancer susceptibility^14^. The rs34330 polymorphism of *p27* gene has also been widely studied for human cancer susceptibility. The existing evidence, however, still remains controversial and has not yet been investigated using meta-analytic methods. Therefore, we aimed in this study to investigate the association of *p27* gene rs34330 polymorphism with cancer susceptibility.

## Results

### Study characteristics

**Figure 1:**
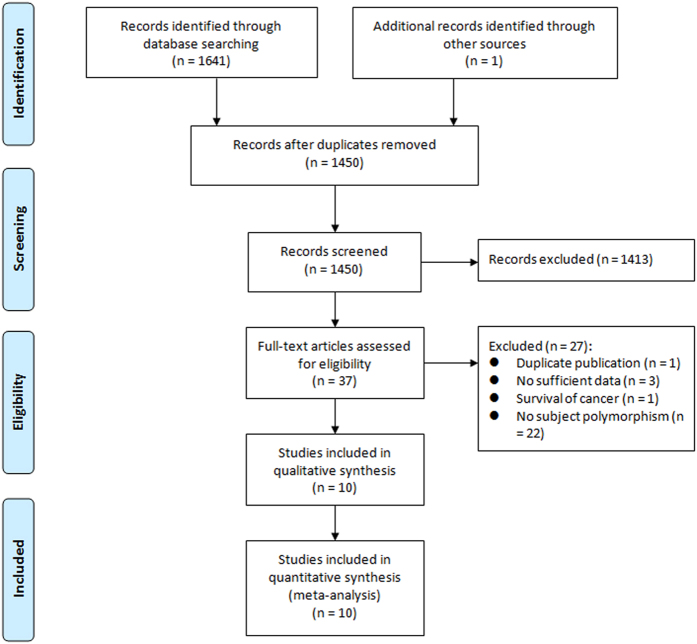
Flow diagram of the study identification and selection process.

Figure 1 summarizes the detailed process of study selection. Based on the search strategy, 1,641 records were retrieved. In this meta-analysis, ten studies^15^,^16^,^17^,^18^,^19^,^20^,^21^,^22^,^23^,^24^ involving 11,214 cases and more than 8,776 controls were identified from the electronic databases according to the inclusion criteria. Characteristics of the identified studies are presented in Table 1. These case-controls studies were published between 2006 and 2014. Of them, three studies were conducted in China, two in the US, one in the UK, one in Australia, one in Turkey, one in Spain, and one in Brazil. Six types of malignant diseases were involved, shown as follows: breast cancer, lung cancer, bladder cancer, thyroid cancer, endometrial cancer, and hepatocellular cancer. The sample size in these studies ranged from 143 to 9,030 individuals. The genotype distributions of cases and controls were presented in seven studies^15^,^16^,^17^,^18^,^20^,^23^,^24^. Other three studies^19^,^21^,^22^ only reported the ORs with 95% CIs in more than two of genetic models, such as homozygous model, heterozygous model, recessive model, or allele model; of these three studies, one study^19^ reported ORs and 95% CIs in two different populations, which was treated as two distinct reports in the combined analysis. All studies were consistent with HWE in controls except one study that did not provide the genotype distribution of controls or report any information for HWE.

### Quantitative analysis

**Figure 2:**
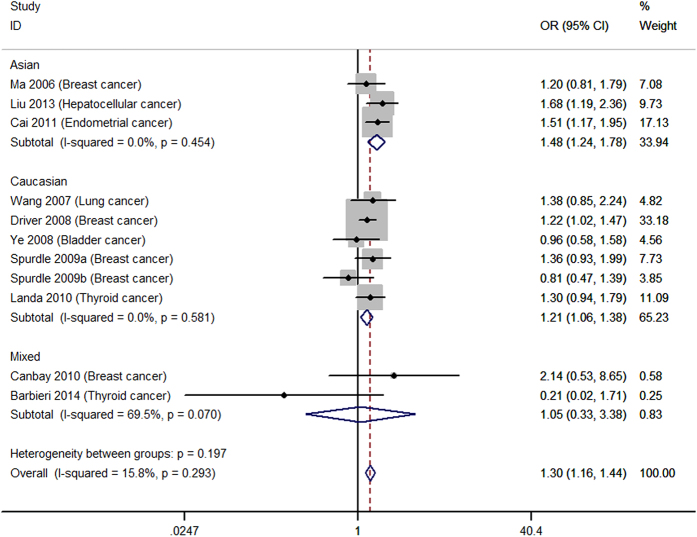
Forest plot for the homozygous model (TT vs. CC). a = BRCA1 carriers; b = BRCA2 carriers.

**Figure 3:**
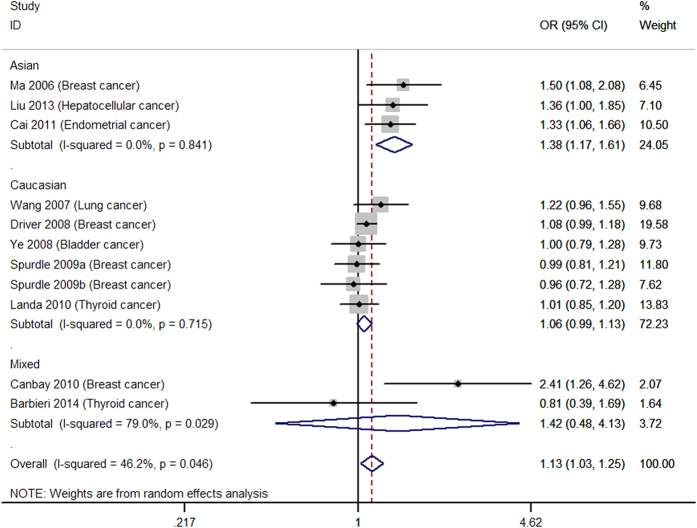
Forest plot for the heterozygous model (CT vs. CC). a = BRCA1 carriers; b = BRCA2 carriers.

**Figure 4:**
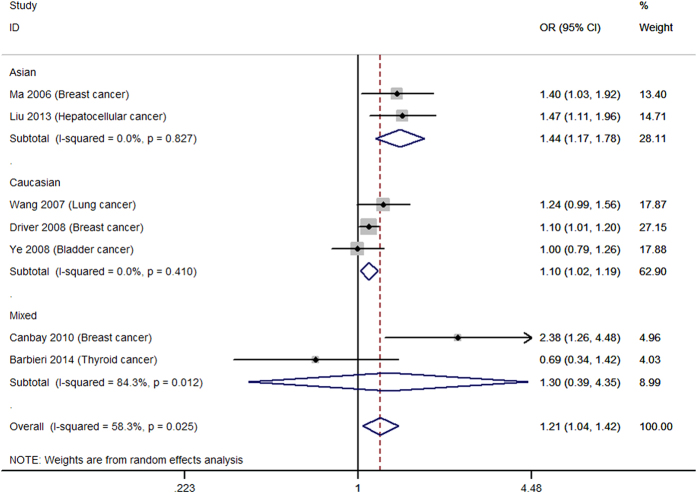
Forest plot for the dominant model (TT + CT vs. CC).

**Figure 5:**
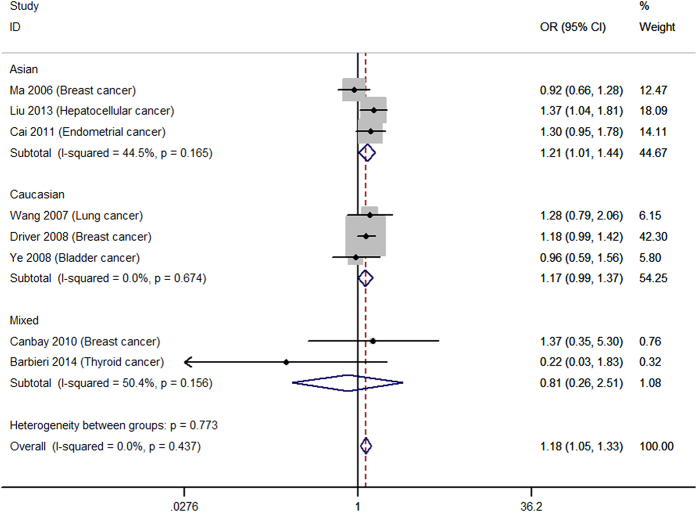
Forest plot for the recessive model (TT vs. CT + CC).

**Figure 6:**
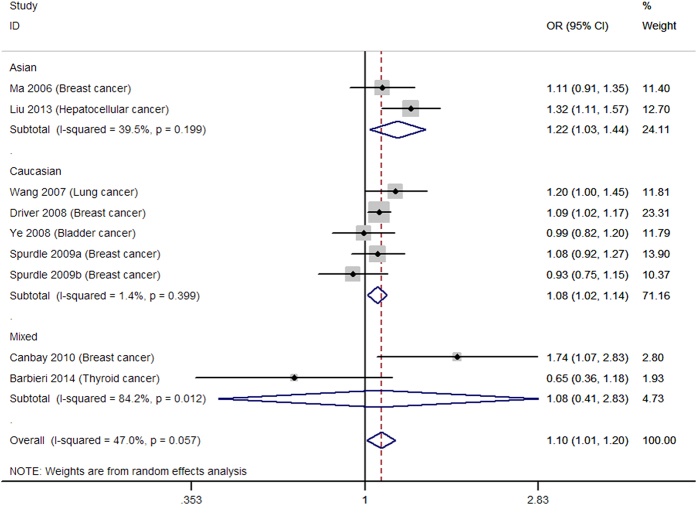
Forest plot for the allele model (T vs. C). a = BRCA1 carriers; b = BRCA2 carriers

Table 2 shows the main results of summarized ORs and 95% CIs for all genetic models estimated in the present analysis of *p27* rs34330 polymorphism and cancer susceptibility. Overall, significantly increased cancer susceptibility was observed in all the tested genetic models: homozygous model (TT vs. CC: OR = 1.30, 95% CI = 1.16–1.44, Fig. 2), heterogeneous model (CT vs. CC: OR = 1.13, 95% CI = 1.03–1.25, Fig. 3), dominant model (TT + CT vs. CC: OR = 1.21, 95% CI = 1.04–1.42, Fig. 4), recessive model (TT vs. CT + CC: OR = 1.18, 95% CI = 1.05–1.33, Fig. 5), allele model (T vs. C: OR = 1.10, 95% CI = 1.01–1.20, Fig. 6). Low to moderate between study heterogeneity was detected (I^2 = ^15.8%, P = 0.293 for TT vs. CC; I^2 = ^46.2%, P = 0.046 for CT vs. CC; I^2^ = 58.3%, P = 0.025 for TT + CT vs. CC; I^2 = ^7.7%, P = 0.369 for TT vs. CT + CC; I^2 = ^47.0%, P = 0.057 for T vs. C).

Subgroup analysis showed increased cancer susceptibility under all tested genetic models (TT vs. CC: OR = 1.48, 95% CI = 1.24–1.78, Fig. 2; CT vs. CC: OR = 1.38, 95% CI = 1.17–1.61, Fig. 3; TT + CT vs. CC: OR = 1.44, 95% CI = 1.17–1.78, Fig. 4; TT vs. CT + CC: OR = 1.21, 95% CI = 1.01–1.44, Fig. 5; T vs. C: OR = 1.22, 95% CI = 1.03–1.44, Fig. 6) for Asians and under three genetic models for Caucasians (TT vs. CC: OR = 1.21, 95% CI = 1.06–1.38, Fig. 2; TT + CT vs. CC: OR = 1.10, 95% CI = 1.02–1.19; T vs. C: OR = 1.08, 95% CI = 1.02–1.14) (Table 2). Pooled results of studies with controls in HWE were similar to the overall results. Sensitivity analysis was performed by removing the study of Canbay 2009 and the results were similar to the overall results under all the genetic models (Table 2).

### Publication bias

**Figure 7:**
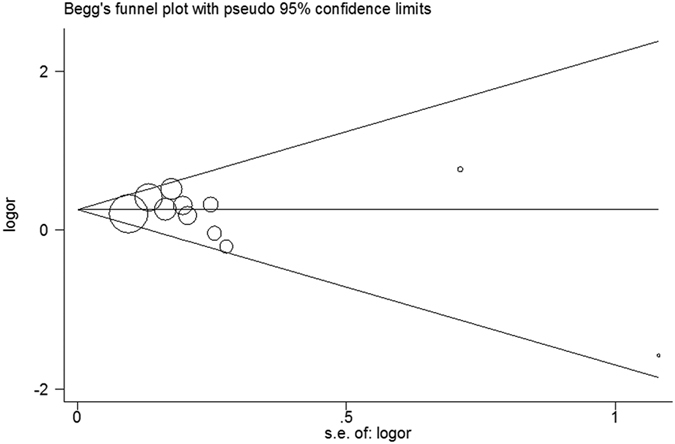
Begg’s funnel plot based on homozygous model (TT vs. CC).

According to funnel plots and Egger’s test, no publication bias was observed in this meta-analysis (homozygous model: P = 0.339 for Egger’s test, Fig. 7).

## Discussion

As single studies may have inadequate statistical power to precisely estimate the association between *p27* gene rs34330 polymorphism and cancer susceptibility, we performed this meta-analysis, which is a quantitative approach, to precisely estimate the true effects of gene polymorphism on cancer susceptibility^25^,^26^. The present meta-analysis included ten case-control studies involving 11 214 cancers and more than 8776 controls and suggested that *p27* gene rs34330 polymorphism may increase the susceptibility to cancer, especially in Asian populations.

Cyclins and CDKs play crucial role in the cell cycle during cellular proliferation. These proteins regulate transitions between G_1_, S, G_2_ and M phases of the cell cycle, especially the transition from G_1_ to S^27^,^28^. Results from experimental studies suggested that CDKIs could inhibit cellular proliferation through suppressing the kinase activity of the cyclin-CDK complexes and block the transition from G_1_ to S^29^. Therefore, CDKIs have been identified as tumor suppressor proteins. The *p27*, which is a member of the CDKIs, has been postulated as a tumor suppressor gene^27^. In 2012, a meta-analysis has been performed to investigate the association between rs2066827 polymorphism of *p27* gene and cancer susceptibility, and they suggested that the *p27* gene rs2066827 polymorphism did not associate with the overall cancer susceptibility in the general population^14^. No meta-analysis was conducted investigating the association between other polymorphisms of *p27* gene and cancer susceptibility.

It has been postulated that the rs34330 polymorphism, located in the 5′-untranslating region of *p27* gene, might be correlated with a reduced production of p27 protein^16^; and certain evidence indicated that rs34330 polymorphism could alter the transcription of p27^21^. In recent decades, this mutation has been widely investigated concerning human cancer susceptibility and the current evidence is still inconclusive. Therefore, we performed the present meta-analysis, for the first time to the best of our knowledge, to summarize the true association between *p27* gene rs34330 polymorphism and cancer susceptibility, and we found an elevated risk of cancer development associated with this polymorphism for overall populations. In this meta-analysis, certain evidence of low to moderate degree of between-study heterogeneity was detected in three genetic models (heterozygous, dominant and allele model) and no evidence of heterogeneity was found in homozygous model and recessive model. The low to moderate between-study heterogeneity indicated acceptable credibility of the results of this meta-analysis. Moreover, the results of sensitivity analysis were robust when we excluded the studies with controls not in HWE. The results of subgroup analysis according to ethnicity showed that the elevated risk associated with *p27* gene rs34330 polymorphism were more predominant in Asians than Caucasians. The results indicated that ethnicity might play an important role in cancer susceptibility; however the underlying mechanism is unclear.

Previously, two meta-analyses investigated the association of CDKN1B gene polymorphisms (including rs34330 and rs2066827) and CDKN1B rs2066827 polymorphism with susceptibility to breast cancer, respectively^30^,^31^. The previous meta-analyses found that *p27* gene rs2066827 polymorphism was not associated with breast cancer susceptibility. One meta-analysis^30^ comprehensively evaluated the association between polymorphisms of *p27* gene and breast cancer susceptibility, and they found that *p27* gene rs34330 polymorphism might be associated with breast cancer susceptibility. In this previous meta-analysis^30^, the included studies with rs34330 polymorphism were four case-control studies, which were also included in our meta-analysis. Moreover, due to the quantity of included studies on different type of cancer, we did not performed subgroup analysis according to type of cancer.

In this study, certain limitations should be taken into consideration. First, confounding factors, such as selection bias and measurement bias, might distort the credibility of the result^32^. Second, this meta-analysis only included Asian and Caucasian populations. Therefore, the external validity is relatively limited. Third, we could not eliminate the possibility of publication bias even though we detected no evidence of publication bias through Begg’s funnel plot and Egger’s linear regression method. In addition, the sample size and number of included studies is relatively small for gene-susceptibility investigation.

In conclusion, our meta-analysis suggests that the *p27* gene rs34330 polymorphism might increase the cancer susceptibility, especially in Asians. Further well-designed and large sample size studies are warranted to verify the conclusion.

## Methods

### Literature search

A thorough literature search was performed using PubMed database up to June 2015. We used the following search terms to identify all potential relevant studies: (p27 OR p27Kip1 OR CDKN1B OR “cyclin-dependent kinase inhibitor 1B”) AND (cancer OR carcinoma OR adenocarcinoma OR neoplasm OR tumor) AND (gene OR allele OR polymorphism OR variation OR variant OR mutation). In addition, reference lists of retrieved articles were manually searched. No restriction was applied.

### Inclusion criteria

All the studies included in the meta-analysis should meet the following inclusion criteria: (1) study design was case-control or cohort; (2) studies examined the association between *p27* rs34330 polymorphism and cancer susceptibility; (3) studies presented detailed genotype counts or odds ratios (ORs) with corresponding 95% confidence intervals (CIs).

### Data extraction

Two reviewers independently extracted the following information from all identified studies according to a standardized data collection form: author name, publication year, ethnicity, location, number of cases and controls, type of cancer, source of control, Hardy-Weinberg equilibrium (HWE) in controls, genotyping method, genetic data, and ORs with corresponding 95% CIs. Any disagreements were resolved by discussion.

### Statistical analysis

The OR and 95% CI were used to measure the association between *p27* rs34330 polymorphism and cancer susceptibility. The significance of pooled ORs was determined by Z test at the P < 0.05 level of significance. We estimated the effect of *p27* rs34300 polymorphism on cancer susceptibility using homozygous, heterogeneous, dominant, recessive, and allele models. Heterogeneity test was performed with the use of Q statistic at the P < 0.10 level of significance since the chi-squire test has low power in the situation of a meta-analysis when numbers or sample sizes of included studies were small^33^. We also calculated the I^2^ metric, a quantitative measurement of heterogeneity among studies^33^. The summary ORs were pooled using a fixed-effects model when the studies included were homogeneous (P ≥ 0.1) and a random-effects model when statistical heterogeneity was detected (P < 0.1). Prespecified subgroup analyses according to HWE in controls, ethnicity, and source of control were performed to examine the impacts of these factors. Sensitivity analysis was performed by removing study which was an outlier. Potential publication bias was explored by Begg’s funnel plot and Egger linear regression test^34^. HWE in controls was examined using goodness-of-fit χ^2^ test. All analyses were performed using Stata 12.0 (Stata, College Station, TX). The two-sided P values less than 0.05 were considered statistically significant, except where extra specified.

## Additional Information

**How to cite this article:** Cheng, X.-K. *et al*. Genetic association between the cyclin-dependent kinase inhibitor gene* p27/Kip1* polymorphism (rs34330) and cancer susceptibility: a meta-analysis. *Sci. Rep.* **7**, 44871; doi: 10.1038/srep44871 (2017).

**Publisher's note:** Springer Nature remains neutral with regard to jurisdictional claims in published maps and institutional affiliations.

## Figures and Tables

**Table 1:** Characteristics of included studies in the meta-analysis.

Reference	Country	Ethnicity	Cancer type	Sample size (case/control)	Control source	Genotyping method	HWE in controls
Ma^15^	China	Asian	Breast cancer	368/476	Population based	PIRA-PCR	Yes
Wang^16^	USA	Caucasian	Lung cancer	1518/1518	Hospital based	TaqMan	Yes
Driver^17^	UK	Caucasian	Breast cancer	4470/4560	Population based	TaqMan	Yes
Ye^18^	USA	Caucasian	Bladder cancer	591/602	Hospital based	PCR-RFLP	Yes
Spurdle^19^	Australia	Caucasian	Breast cancer	2359/NR	Hospital based	PCR	NA
Canbay^20^	Turkey	Mixed	Breast cancer	78/84	Population based	PCR-RFLP	Yes
Landa^21^	Spain	Caucasian	Thyroid cancer	649/385	Population based	qRT-PCR	Yes
Cai^22^	China	Asian	Endometrial cancer	1028/1003	Population based	PCR	Yes
Liu^23^	China	Asian	Hepatocellular cancer	476/526	Hospital based	MALDI-TOF	Yes
Barbieri^24^	Brazil	Mixed	Thyroid cancer	45/98	Population based	TaqMan	Yes

HWE = Hardy-Weinberg equilibrium; NA = not available.

**Table 2:** Meta-analysis of all studies and subgroups.

Overall and subgroups	Number of Studies	Heterogeneity		Model	Meta-analysis	
		*P*	*I*^2^ (%)		OR (95%CI)	P for OR
**Homozygous model (TT vs. CC)**						
Overall	10	0.293	15.8	Fixed	1.30 (1.16–1.44)	<0.001
HWE (Yes)	9	0.354	9.7	Fixed	1.32 (1.18–1.47)	<0.001
Sensitivity analysis^a^	9	0.251	20.9	Fixed	1.29 (1.16–1.44)	<0.001
Asians	3	0.454	0.0	Fixed	1.48 (1.24–1.78)	<0.001
Caucasians	5	0.581	0.0	Fixed	1.21 (1.06–1.38)	0.004
Mixed	2	0.07	69.5	Fixed	1.05 (0.33–3.38)	0.928
Hospital-based	4	0.159	39.3	Fixed	1.30 (1.07–1.57)	0.008
Population-based	6	0.381	5.6	Fixed	1.30 (1.14–1.47)	<0.001
**Heterozygous model (CT vs. CC)**						
Overall	10	0.046	46.2	Random	1.13 (1.03–1.25)	0.013
HWE (Yes)	9	0.039	50.9	Random	1.18 (1.05–1.32)	0.005
Sensitivity analysis^a^	10	0.164	30.7	Fixed	1.10 (1.03–1.16)	0.003
Asians	3	0.841	0.0	Random	1.38 (1.17–1.61)	<0.001
Caucasians	5	0.715	0.0	Random	1.06 (0.99–1.13)	0.096
Mixed	2	0.029	79.0	Random	1.42 (0.48–4.13)	0.525
Hospital-based	4	0.299	18.2	Random	1.08 (0.95–1.22)	0.219
Population-based	6	0.020	62.7	Random	1.20 (1.02–1.41)	0.031
**Dominant model (TT + CT vs. CC)**						
Overall	7	0.025	58.3	Random	1.21 (1.04–1.42)	0.014
HWE (Yes)	7	0.025	58.3	Random	1.21 (1.04–1.42)	0.014
Sensitivity analysis^a^	6	0.103	45.3	Fixed	1.13 (1.05–1.21)	0.001
Asians	2	0.827	0.0	Random	1.44 (1.17–1.78)	0.001
Caucasians	3	0.410	0.0	Random	1.10 (1.02–1.19)	0.012
Mixed	2	0.012	84.3	Random	1.30 (0.39–4.35)	0.669
Hospital-based	3	0.104	55.8	Random	1.21 (0.97–1.50)	0.085
Population-based	4	0.025	68.0	Random	1.26 (0.92–1.72)	0.157
**Recessive model (TT vs. CT + CC)**						
Overall	8	0.437	0.0	Fixed	1.18 (1.05–1.33)	0.006
HWE (Yes)	8	0.437	0.0	Fixed	1.18 (1.05–1.33)	0.006
Sensitivity analysis^a^	7	0.333	12.7	Fixed	1.18 (1.05–1.33)	0.006
Asians	3	0.165	44.5	Fixed	1.21 (1.01–1.44)	0.036
Caucasians	3	0.674	0.0	Fixed	1.17 (0.99–1.37)	0.058
Mixed	2	0.156	50.4	Fixed	0.81 (0.26–2.51)	0.709
Hospital-based	3	0.453	0.0	Fixed	1.26 (1.02–1.57)	0.003
Population-based	5	0.309	16.6	Fixed	1.15 (1.00–1.32)	0.057
**Allele model (T vs. C)**						
Overall	8	0.057	47.0	Random	1.10 (1.01–1.20)	0.05
HWE (Yes)	7	0.054	51.5	Random	1.14 (1.02–1.26)	0.018
Sensitivity analysis^a^	7	0.115	39.6	Fixed	1.09 (1.04–1.15)	0.001
Asians	2	0.199	39.5	Random	1.22 (1.03–1.44)	0.023
Caucasians	4	0.399	1.4	Random	1.08 (1.02–1.14)	0.010
Mixed	2	0.012	84.2	Random	1.08 (0.41–2.83)	0.871
Hospital-based	4	0.073	53.3	Random	1.10 (0.98–1.24)	0.115
Population-based	4	0.089	53.9	Random	1.11 (0.93–1.32)	0.251

^a^Removing the study of Canbay 2009.

HWE = Hardy-Weinberg equilibrium; OR = odds ratio; CI = confidence interval.
